# Supplementary material for: Screening for Influenza and Morbillivirus in Seals and Porpoises in the Baltic and North Sea
Source: Pathogens. 2023 Feb 21;12(3):357. doi: 10.3390/pathogens12030357 (PMC10054458; doi:10.3390/pathogens12030357)
Supplement: Supplementary file 1 [file pathogens-12-00357-s001.zip › pathogens-2142818-supplementary.pdf]

**SUPPLEMENTARY MATERIAL**

**Supplementary Table S1**

| Sample number | Species | Collection date | Sex | Age | Country | Area | Origin of animal | Swab | Tissue | Extraction |
|---|---|---|---|---|---|---|---|---|---|---|
| HG-SE-001 | Grey seal | 2002-12-28 | Male | 3 | Sweden | Arkona Basin | Found dead | | + | IL |
| HG-SE-002 | Grey seal | 2002-10-25 | Male | 14 | Sweden | Baltic proper | Shot | | + | IL |
| HG-SE-003 | Grey seal | 2002-05-10 | Female | 13 | Sweden | Bothnian Bay | Shot | | + | IL |
| HG-SE-004 | Grey seal | 2002-11-03 | Female | 3 | Sweden | Bothnian Bay | Shot | | + | IL |
| HG-SE-005 | Grey seal | 2002-04-25 | Female | 0-1 | Sweden | Bothnian Sea | Bycaught | | + | IL |
| HG-SE-006 | Grey seal | 2002-08-20 | Female | 0-1 | Sweden | Bothnian Sea | Shot | | + | IL |
| HG-SE-007 | Grey seal | 2002-10-21 | Female | 11 | Sweden | Bothnian Sea | Bycaught | | + | IL |
| HG-SE-008 | Grey seal | 2002-04-03 | Male | 6 | Sweden | Skagerrak | Found dead | | + | IL |
| HG-SE-009 | Grey seal | 2003-05-25 | Female | 5 | Sweden | Baltic proper | Bycaught | | + | IL |
| HG-SE-010 | Grey seal | 2003-11-16 | Male | 32 | Sweden | Baltic proper | Bycaught | | + | IL |
| HG-SE-011 | Grey seal | 2003-04-22 | Male | 0-1 | Sweden | Bornholm Basin | Bycaught | | + | IL |
| HG-SE-012 | Grey seal | 2003-05-04 | Male | 3 | Sweden | Bothnian Sea | Bycaught | | + | IL |
| HG-SE-013 | Grey seal | 2003-07-08 | Female | 3 | Sweden | Bothnian Sea | Shot | | + | IL |
| HG-DK-001 | Grey seal | 2010-06-26 | Male | Young | Denmark | Kattegat | Shot | | + | PL |
| HG-DK-002 | Grey seal | 2010-03-03 | Male | Adult | Denmark | The Sound | Found dead | | + | PL |
| HG-DK-003 | Grey seal | 2012-08-12 | Male | - | Denmark | SW Baltic | Shot | | + | PL |
| HG-SE-014 | Grey seal | 2013-04-21 | Female | 3 | Sweden | Baltic proper | Shot | | + | IL |
| HG-SE-015 | Grey seal | 2013-08-07 | Male | 0 | Sweden | Baltic proper | Entanglement | | + | IL |
| HG-SE-016 | Grey seal | 2013-05-11 | Female | 0 | Sweden | Bothnian Bay | Shot | | + | IL |
| HG-SE-017 | Grey seal | 2013-07-25 | Female | 5 | Sweden | Bothnian Bay | Shot | | + | IL |
| HG-SE-018 | Grey seal | 2013-04-27 | Male | 17 | Sweden | Bothnian Sea | Shot | | + | IL |
| HG-SE-019 | Grey seal | 2013-08-01 | Female | 6 | Sweden | Bothnian Sea | Shot | | + | IL |
| HG-DK-004 | Grey seal | 2014-01-15 | - | - | Denmark | Arkona Basin | Found dead | | + | PL |
| HG-DK-005 | Grey seal | 2014-11-22 | Male | - | Denmark | Arkona Basin | Found dead | | + | PL |

| Sample number | Species | Collection date | Sex | Age | Country | Area | Origin of animal | Swab | Tissue | Extraction |
|---|---|---|---|---|---|---|---|---|---|---|
| HG-SE-020 | Grey seal | 2014-04-25 | Female | 19 | Sweden | Baltic proper | Shot | | + | IL |
| HG-SE-021 | Grey seal | 2014-08-25 | Female | 3 | Sweden | Baltic proper | Entanglement | | + | IL |
| HG-SE-022 | Grey seal | 2014-12-31 | Male | 2 | Sweden | Bornholm Basin | Shot | | + | IL |
| HG-SE-023 | Grey seal | 2014-12-31 | Male | 2 | Sweden | Bornholm Basin | Shot | | + | IL |
| HG-SE-024 | Grey seal | 2014-05-11 | Male | 8 | Sweden | Bothnian Bay | Shot | | + | IL |
| HG-SE-025 | Grey seal | 2014-08-24 | Male | 10 | Sweden | Bothnian Bay | Shot | | + | IL |
| HG-SE-026 | Grey seal | 2014-07-19 | Male | 4 | Sweden | Bothnian Sea | Shot | | + | IL |
| HG-SE-027 | Grey seal | 2014-10-24 | Male | 2 | Sweden | Bothnian Sea | Entanglement | | + | IL |
| HG-DK-006 | Grey seal | 2014-05-02 | - | Pub | Denmark | Danish Straits | Found dead | | + | PL |
| HG-DK-007 | Grey seal | 2014-04-23 | Female | Adult | Denmark | SW Baltic | Found dead | | + | PL |
| HG-DK-008 | Grey seal | 2014-11-22 | Male | Adult | Denmark | The Sound | Found dead | | + | PL |
| HG-DK-009 | Grey seal | 2014-03-02 | Female | Juvenile | Denmark | West coast | Shot | | + | PL |
| HG-SE-028 | Grey seal | 2015-04-28 | Male | 7 | Sweden | Baltic proper | Shot | | + | IL |
| HG-SE-029 | Grey seal | 2015-05-08 | Male | 5 | Sweden | Bornholm Basin | Shot | | + | IL |
| HG-SE-030 | Grey seal | 2015-08-13 | Female | 8 | Sweden | Bornholm Basin | Shot | | + | IL |
| HG-SE-031 | Grey seal | 2015-11-03 | Male | 12 | Sweden | Bornholm Basin | Shot | | + | IL |
| HG-SE-032 | Grey seal | 2015-05-08 | Male | 18 | Sweden | Bothnian Bay | Shot | | + | IL |
| HG-SE-033 | Grey seal | 2015-04-24 | Female | - | Sweden | Bothnian Sea | Shot | + | + | SP, IL |
| HG-SE-034 | Grey seal | 2015-04-24 | - | - | Sweden | Bothnian Sea | Shot | + | + | SP, IL |
| HG-SE-035 | Grey seal | 2015-04-25 | Male | - | Sweden | Bothnian Sea | Shot | + | + | SP, IL |
| HG-SE-036 | Grey seal | 2015-04-25 | Male | - | Sweden | Bothnian Sea | Shot | + | + | SP, IL |
| HG-SE-037 | Grey seal | 2015-07-15 | Female | 2 | Sweden | Bothnian Sea | Shot | | + | IL |
| HG-SE-038 | Grey seal | 2016-09-19 | Female | - | Sweden | Bothnian Sea | Shot | + | + | SP, IL |
| HG-PL-001 | Grey seal | 2017-07-12 | Female | Yearling | Poland | Bornholm Basin | Euthanized | + | + | SP, IL |

| Sample number | Species | Collection date | Sex | Age | Country | Area | Origin of animal | Swab | Tissue | Extraction |
|---|---|---|---|---|---|---|---|---|---|---|
| HG-SE-039 | Grey seal | 2018-09-02 | Female | Adult | Sweden | Arkona Basin | Shot | + | + | SP, IS |
| HG-SE-040 | Grey seal | 2018-09-02 | Male | Adult | Sweden | Arkona Basin | Shot | + | + | SP, IL |
| HG-SE-041 | Grey seal | 2018-09-02 | Female | Adult | Sweden | Arkona basin | Shot | + | + | SP, IL |
| HG-PL-002 | Grey seal | 2018-03-16 | Male | Adult | Poland | Baltic proper | Found dead | + | + | SP, IL |
| HG-PL-003 | Grey seal | 2018-04-05 | Male | Pup | Poland | Baltic proper | Euthanized | | + | IL |
| HG-PL-004 | Grey seal | 2018-06-06 | Female | Yearling | Poland | Baltic proper | Found dead | + | | SP |
| HG-PL-005 | Grey seal | 2018-06-14 | Female | Yearling | Poland | Baltic proper | Found dead | + | + | SP, IL |
| HG-SE-042 | Grey seal | 2018-08-05 | Male | Juvenile | Sweden | Baltic proper | Shot | + | | SP, IL |
| HG-SE-043 | Grey seal | 2018-08-05 | Male | Adult | Sweden | Baltic proper | Shot | + | + | SP, IL |
| HG-SE-044 | Grey seal | 2018-08-05 | Male | Juvenile | Sweden | Baltic proper | Shot | + | + | SP, IL |
| HG-SE-045 | Grey seal | 2018-08-05 | Male | Juvenile | Sweden | Baltic proper | Shot | + | + | SP, IL |
| HG-SE-046 | Grey seal | 2018-08-05 | Male | Juvenile | Sweden | Baltic proper | Shot | + | + | SP, IL |
| HG-SE-047 | Grey seal | 2018-08-05 | Female | Juvenile | Sweden | Baltic proper | Shot | | + | IL |
| HG-SE-048 | Grey seal | 2018-08-05 | Male | Juvenile | Sweden | Baltic proper | Shot | + | + | SP, IL |
| HG-SE-049 | Grey seal | 2018-08-06 | Male | Adult | Sweden | Baltic proper | Shot | + | | SP, IL |
| HG-SE-050 | Grey seal | 2018-08-07 | Male | Juvenile | Sweden | Baltic proper | Shot | + | + | SP, IL |
| HG-SE-051 | Grey seal | 2018-08-07 | Female | Juvenile | Sweden | Baltic proper | Shot | + | + | SP, IL |
| HG-SE-052 | Grey seal | 2018-08-29 | Male | - | Sweden | Baltic proper | Shot | + | + | SP, IL |
| HG-SE-053 | Grey seal | 2018-08-31 | Male | Juvenile | Sweden | Baltic proper | Shot | + | + | SP, IL |
| HG-SE-054 | Grey seal | 2018-09-05 | Female | - | Sweden | Baltic proper | Shot | | + | IL |
| HG-SE-055 | Grey seal | 2018-09-13 | Male | - | Sweden | Baltic proper | Shot | | + | IL |
| HG-SE-056 | Grey seal | 2018-09-19 | Male | Juvenile | Sweden | Baltic proper | Shot | | + | IL |
| HG-PL-006 | Grey seal | 2018-10-25 | Male | Yearling | Poland | Baltic proper | Euthanized | + | + | SP, IL |
| HG-SE-057 | Grey seal | 2018-?-? | Female | - | Sweden | Baltic proper | Shot | + | + | SP, IL |

| Sample number | Species | Collection date | Sex | Age | Country | Area | Origin of animal | Swab | Tissue | Extraction |
|---|---|---|---|---|---|---|---|---|---|---|
| HG-SE-058 | Grey seal | 2018-?-? | Female | Juvenile | Sweden | Baltic proper | Shot | + | + | SP, IL |
| HG-PL-007 | Grey seal | 2018-05-01 | Male | Yearling | Poland | Bornholm Basin | Found dead | + | + | SP, IL |
| HG-SE-059 | Grey seal | 2018-08-05 | Male | - | Sweden | Bornholm Basin | Shot | + | + | SP, IL |
| HG-SE-060 | Grey seal | 2018-08-29 | Female | Adult | Sweden | Bornholm Basin | Shot | + | + | SP, IL |
| HG-SE-061 | Grey seal | 2018-09-07 | Female | - | Sweden | Bornholm Basin | Shot | + | + | SP, IL |
| HG-SE-062 | Grey seal | 2018-08-26 | Male | Adult | Sweden | Borrnholm Basin | Shot | + | + | SP, IL |
| HG-SE-063 | Grey seal | 2018-09-02 | - | - | Sweden | Bothnian Bay | Shot | + | + | SP, IL |
| HG-SE-064 | Grey seal | 2018-09-02 | Female | Juvenile | Sweden | Bothnian Bay | Bycaught | + | + | SP, IL |
| HG-SE-065 | Grey seal | 2018-08-02 | Female | Juvenile | Sweden | Bothnian Sea | Shot | + | + | SP, IL |
| HG-SE-066 | Grey seal | 2018-08-02 | Male | Juvenile | Sweden | Bothnian Sea | Shot | + | + | SP, IL |
| HG-SE-067 | Grey seal | 2018-08-02 | - | - | Sweden | Bothnian Sea | Shot | + | + | SP, IL |
| HG-SE-068 | Grey seal | 2018-08-03 | Male | Subadult | Sweden | Bothnian Sea | Shot | + | + | SP, IL |
| HG-SE-069 | Grey seal | 2018-08-03 | Female | Adult | Sweden | Bothnian Sea | Shot | + | + | SP, IL |
| HG-SE-070 | Grey seal | 2018-08-04 | Female | Adult | Sweden | Bothnian Sea | Shot | + | + | SP, IL |
| HG-SE-071 | Grey seal | 2018-08-04 | Female | Adult | Sweden | Bothnian Sea | Shot | + | + | SP, IL |
| HG-SE-072 | Grey seal | 2018-08-10 | Male | - | Sweden | Bothnian Sea | Shot | + | + | SP, IL |
| HG-SE-073 | Grey seal | 2018-08-14 | Male | Adult | Sweden | Bothnian Sea | Shot | + | + | SP, IL |
| HG-SE-074 | Grey seal | 2018-08-14 | Female | Adult | Sweden | Bothnian Sea | Shot | + | + | SP, IL |
| HG-SE-075 | Grey seal | 2018-08-14 | Female | Adult | Sweden | Bothnian Sea | Shot | + | + | SP, IL |
| HG-SE-076 | Grey seal | 2018-08-15 | Male | Adult | Sweden | Bothnian Sea | Shot | + | + | SP, IL |
| HG-SE-077 | Grey seal | 2018-08-15 | Female | Juvenile | Sweden | Bothnian Sea | Shot | + | + | SP, IL |
| HG-SE-078 | Grey seal | 2018-08-15 | Male | - | Sweden | Bothnian Sea | Shot | + | + | SP, IL |
| HG-SE-079 | Grey seal | 2018-08-15 | Female | Juvenile | Sweden | Bothnian Sea | Shot | + | + | SP, IL |
| HG-SE-080 | Grey seal | 2018-08-20 | Male | Adult | Sweden | Bothnian Sea | Shot | + | + | SP, IL |

| Sample number | Species | Collection date | Sex | Age | Country | Area | Origin of animal | Swab | Tissue | Extraction |
|---|---|---|---|---|---|---|---|---|---|---|
| HG-SE-081 | Grey seal | 2018-08-20 | Female | Adult | Sweden | Bothnian Sea | Shot | + | + | SP, IL |
| HG-SE-082 | Grey seal | 2018-08-22 | Female | Adult | Sweden | Bothnian Sea | Shot | + | + | SP, IL |
| HG-SE-083 | Grey seal | 2018-08-22 | Male | Adult | Sweden | Bothnian Sea | Shot | + | + | SP, IL |
| HG-SE-084 | Grey seal | 2018-08-22 | Female | Juvenile | Sweden | Bothnian Sea | Shot | + | + | SP, IL |
| HG-SE-085 | Grey seal | 2018-08-22 | Female | Adult | Sweden | Bothnian Sea | Shot | + | + | SP, IL |
| HG-SE-086 | Grey seal | 2018-08-24 | Female | Adult | Sweden | Bothnian Sea | Shot | + | + | SP, IL |
| HG-SE-087 | Grey seal | 2018-08-25 | Female | Adult | Sweden | Bothnian Sea | Shot | + | + | SP, IL |
| HG-SE-088 | Grey seal | 2018-08-25 | Female | Adult | Sweden | Bothnian Sea | Bycaught | + | + | SP, IL |
| HG-SE-089 | Grey seal | 2018-09-02 | Male | Adult | Sweden | Bothnian Sea | Shot | + | + | SP, IL |
| HG-SE-090 | Grey seal | 2018-09-02 | Female | Juvenile | Sweden | Bothnian Sea | Shot | + | + | SP, IL |
| HG-SE-091 | Grey seal | 2018-09-02 | Female | Juvenile | Sweden | Bothnian Sea | Shot | + | + | SP, IL |
| HG-SE-092 | Grey seal | 2018-09-04 | Female | Adult | Sweden | Bothnian Sea | Shot | + | + | SP, IL |
| HG-SE-093 | Grey seal | 2018-09-04 | Female | Adult | Sweden | Bothnian Sea | Shot | + | + | SP, IL |
| HG-SE-094 | Grey seal | 2018-09-04 | Female | Adult | Sweden | Bothnian Sea | Shot | + | + | SP, IL |
| HG-SE-095 | Grey seal | 2018-09-04 | Female | Adult | Sweden | Bothnian Sea | Shot | + | + | SP, IL |
| HG-SE-096 | Grey seal | 2018-09-07 | Female | - | Sweden | Bothnian Sea | Shot | + | + | SP, IL |
| HG-SE-097 | Grey seal | 2018-09-13 | Male | Adult | Sweden | Bothnian Sea | Shot | + | + | SP, IL |
| HG-SE-098 | Grey seal | 2018-10-01 | Female | Juvenile | Sweden | Bothnian Sea | Shot | + | + | SP, IL |
| HG-SE-099 | Grey seal | 2018-10-20 | Male | - | Sweden | Bothnian Sea | Shot | + | + | SP, IL |
| HG-GE-001 | Grey seal | 2018-07-31 | Female | Adult | Germany | North Sea | Shot | + | + | SP, IL |
| HG-DK-010 | Grey seal | 2019-02-05 | Female | Adult | Denmark | Arkona Basin | Shot | + | + | SP, IL |
| HG-DK-011 | Grey seal | 2019-10-01 | Male | Adult | Denmark | Arkona Basin | Shot | + | + | SP, IL |
| HG-SE-100 | Grey seal | 2019-08-03 | Male | - | Sweden | Baltic proper | Bycaught |  | + | IL |
| HG-SE-101 | Grey seal | 2019-08-15 | Male | - | Sweden | Baltic proper | Bycaught | + | + | SP, IL |

| Sample number | Species | Collection date | Sex | Age | Country | Area | Origin of animal | Swab | Tissue | Extraction |
|---|---|---|---|---|---|---|---|---|---|---|
| HG-SE-102 | Grey seal | 2019-09-04 | Male | - | Sweden | Baltic proper | Bycaught | + | + | SP, IL |
| HG-SE-103 | Grey seal | 2019-09-16 | Female | - | Sweden | Baltic proper | Bycaught | | + | IL |
| HG-SE-104 | Grey seal | 2019-10-15 | Male | - | Sweden | Baltic proper | Shot | + | + | SP, IL |
| HG-SE-105 | Grey seal | 2019-08-10 | Fenale | - | Sweden | Bothnian Sea | Shot | + | + | SP, IL |
| HG-GE-002 | Grey seal | 2019-03-23 | Male | - | Germany | North Sea | Shot | + | + | SP, IL |
| HG-PL-008 | Grey seal | 2019-04-14 | Male | - | Poland | - | Found dead | + | + | SP, IL |

**Supplementary Table S1.** Overview of grey seal samples screened in this study including sample information such as collection date, sex, age, collection area and sample type listed when possible. (-) indicates that there is no available information. The sample type for each extraction is indicated by SP = Pooled extractions of tracheal swabs, IL = Individual extraction from lung tissue, PL = Pooled extractions of lung tissue from 3-5 individuals.

**Supplementary Table S2**

| Sample number | Species | Collection date | Sex | Age | Country | Area | Origin of animal | Swab | Tissue | Extraction |
|---|---|---|---|---|---|---|---|---|---|---|
| PV-SE-001 | Harbour seal | 2002-09-07 | Male | 2 | Sweden | Baltic proper (Kalmarsund) | Found dead | - | + | IL |
| PV-SE-002 | Harbour seal | 2002-07-25 | Male | 4 | Sweden | Skagerrak | Found dead | - | + | IL |
| PV-SE-003 | Harbour seal | 2003-10-14 | Male | - | Sweden | Baltic proper (Kalmarsund) | Bycaught | - | + | IL |
| PV-DK-001 | Harbour seal | 2003-05-27 | Female | 2 years | Denmark | Danish straits | Shot | - | + | PL |
| PV-DK-002 | Harbour seal | 2003-08-04 | Male | - | Denmark | Danish straits | Shot | - | + | PL |
| PV-DK-003 | Harbour seal | 2006-11-15 | Female | 1-2 years | Denmark | Danish straits | Shot | - | + | PL |
| PV-DK-004 | Harbour seal | 2006-09-22 | Female | 2-3 years | Denmark | Kattegat | Bycaught | - | + | PL |
| PV-DK-005 | Harbour seal | 2006-09-22 | Male | 0-1 years | Denmark | Kattegat | Bycaught | - | + | PL |
| PV-DK-006 | Harbour seal | 2007-06-21 | Female | 0 years | Denmark | Kattegat | Found dead | - | + | PL |
| PV-DK-007 | Harbour seal | 2007-06-21 | Male | 1-2 years | Denmark | Kattegat | Found dead | - | + | PL |
| PV-DK-008 | Harbour seal | 2007-06-21 | Male | 1-2 Years | Denmark | Kattegat | Found dead | - | + | PL |
| PV-DK-009 | Harbour seal | 2007-06-21 | Male | > 1 years | Denmark | Kattegat | Found dead | - | + | PL |
| PV-DK-010 | Harbour seal | 2007-06-21 | Female | > 1 years | Denmark | Kattegat | Found dead | - | + | PL |
| PV-DK-011 | Harbour seal | 2007-06-21 | Female | > 1 years | Denmark | Kattegat | Found dead | - | + | PL |
| PV-DK-012 | Harbour seal | 2007-06-21 | Female | > 1 years | Denmark | Kattegat | Found dead | - | + | PL |
| PV-DK-013 | Harbour seal | 2007-06-21 | Female | > 1 years | Denmark | Kattegat | Found dead | - | + | PL |
| PV-DK-014 | Harbour seal | 2007-06-21 | Female | 1 | Denmark | Kattegat | Found dead | - | + | PL |
| PV-DK-015 | Harbour seal | 2008-09-30 | Female | Adult | Denmark | Danish straits | Shot | - | + | PL |
| PV-DK-016 | Harbour seal | 2008-06-16 | Male | 0 years | Denmark | Kattegat | Found dead | - | + | PL |
| PV-DK-017 | Harbour seal | 2010-08-12 | Male | - | Denmark | Arkona Basin | Shot | - | + | PL |
| PV-DK-018 | Harbour seal | 2010-11-21 | Female | Adult | Denmark | Danish straits | NA | - | + | IL |
| PV-DK-019 | Harbour seal | 2010-07-01 | Female | Juvenile/Yearling | Denmark | Kattegat | Found dead | - | + | PL |
| PV-DK-020 | Harbour seal | 2011-05-11 | Male | Adult | Denmark | Danish straits | Shot | - | + | PL |
| PV-DK-021 | Harbour seal | 2012-08-12 | Female | - | Denmark | Arkona Basin | Shot | - | + | PL |
| PV-DK-022 | Harbour seal | 2012-08-12 | Male | Adult | Denmark | Arkona Basin | Shot | - | + | IL |

| Sample number | Species | Collection date | Sex | Age | Country | Area | Origin of animal | Swab | Tissue | Extraction |
|---|---|---|---|---|---|---|---|---|---|---|
| PV-DK-023 | Harbour seal | 2012-09-29 | Female | 2-3 years | Denmark | Arkona Basin | Shot | - | + | PL |
| PV-SE-004 | Harbour seal | 2012-11-13 | Male | 8 | Sweden | Baltic proper | Shot | - | + | IL |
| PV-DK-024 | Harbour seal | 2012-08-22 | Male | Juvenile | Denmark | Danish straits | Shot | - | + | PL |
| PV-DK-025 | Harbour seal | 2012-05-25 | Female | - | Denmark | The Sound | Shot | - | + | PL |
| PV-DK-026 | Harbour seal | 2014-11-24 | Male | - | Denmark | Arkona Basin | Bycaught | - | + | PL |
| PV-SE-005 | Harbour seal | 2014-04-01 | Male | 2 | Sweden | Baltic proper | Entanglement | - | + | IL |
| PV-SE-006 | Harbour seal | 2014-09-21 | Male | 6 | Sweden | Baltic proper | Entanglement | - | + | IL |
| PV-DK-027 | Harbour seal | 2014-09-13 | Male | 0 | Denmark | Kattegat | Found dead | - | + | PL, IL |
| PV-DK-028 | Harbour seal | 2014-08-29 | Female | - | Denmark | Limfjorden | Euthanized | - | + | PL, IL |
| PV-DK-029 | Harbour seal | 2014-08-29 | Male | - | Denmark | Limfjorden | Euthanized | - | + | PL, IL |
| PV-DK-030 | Harbour seal | 2014-08-29 | Male | - | Denmark | Limfjorden | Euthanized | - | + | PL, IL |
| PV-DK-031 | Harbour seal | 2014-08-29 | Female | - | Denmark | Limfjorden | Euthanized | - | + | PL, IL |
| PV-DK-032 | Harbour seal | 2014-02-21 | Female | Juvenile | Denmark | North Sea | Found dead | - | + | PL |
| PV-DK-033 | Harbour seal | 2014-04-28 | Female | Adult | Denmark | North Sea | Shot | - | + | PL |
| PV-DK-034 | Harbour seal | 2014-05-07 | Female | Young | Denmark | The Sound | Shot | - | + | PL |
| PV-DK-035 | Harbour seal | 2014-05-07 | Female | Young | Denmark | The Sound | Shot | - | + | PL |
| PV-DK-036 | Harbour seal | 2014-05-07 | Male | Adult | Denmark | The Sound | Shot | - | + | PL |
| PV-DK-037 | Harbour seal | 2014-08-28 | Female | Young | Denmark | The Sound | Shot | - | + | PL |
| PV-DK-038 | Harbour seal | 2014-08-28 | Female | Adult | Denmark | The Sound | Shot | - | + | PL |
| PV-SE-007 | Harbour seal | 2015-02-03 | Female | 3 | Sweden | The Sound | Entanglement | - | + | IL |
| PV-DK-039 | Harbour seal | 2015-04-07 | Female | Immature | Denmark | The Sound | Shot | - | + | PL |
| PV-DK-040 | Harbour seal | 2016-01-15 | Male | Adult | Denmark | The Sound | Shot | - | + | PL |
| PV-SE-008 | Harbour seal | 2018-10-11 | Male | - | Sweden | Bornholm Basin | Bycaught | - | + | IL |
| PV-DK-041 | Harbour seal | 2018-08-20 | Male | Current year | Denmark | Danish Straits | Bycaught | + | + | SP, IL |
| PV-DK-042 | Harbour seal | 2018-10-29 | Female | Adult | Denmark | Danish Straits | Shot | + | + | SP, IL |

| Sample number | Species | Collection date | Sex | Age | Country | Area | Origin of animal | Swab | Tissue | Extraction |
|---|---|---|---|---|---|---|---|---|---|---|
| PV-GE-001 | Harbour seal | 2018-11-01 | Male | - | Germany | Danish Straits | Euthanized | + | + | SP, IL |
| PV-SE-009 | Harbour seal | 2018-11-21 | Female | Adult | Sweden | Kattegat | Shot | | + | IL |
| PV-SE-010 | Harbour seal | 2018-12-25 | Male | Adult | Sweden | Kattegat | Shot | + | + | SP, IL |
| PV-SE-011 | Harbour seal | 2018-10-01 | Female | Adult | Sweden | Kattegatt | Shot | + | + | SP, IL |
| PV-SE-012 | Harbour seal | 2018-10-01 | Female | 0-1 | Sweden | Kattegatt | Shot | + | + | SP, IL |
| PV-SE-013 | Harbour seal | 2018-10-01 | Male | 0-1 | Sweden | Kattegatt | Shot | + | + | SP, IL |
| PV-SE-014 | Harbour seal | 2018-10-04 | Female | 0-1 | Sweden | Kattegatt | Shot | + | + | SP, IL |
| PV-SE-015 | Harbour seal | 2018-10-04 | Male | 0-1 | Sweden | Kattegatt | Shot | + | + | SP, IL |
| PV-SE-016 | Harbour seal | 2018-10-07 | Female | - | Sweden | Kattegatt | Shot | + | + | SP, IL |
| PV-SE-017 | Harbour seal | 2018-10-07 | Female | 0-1 | Sweden | Kattegatt | Shot | + | + | SP, IL |
| PV-GE-002 | Harbour seal | 2018-01-13 | Female | Adult | Germany | North Sea | Found dead | + | + | SP, IL |
| PV-GE-003 | Harbour seal | 2019-02-20 | Female | Adult | Germany | North Sea | Found dead | | + | IL |
| PV-GE-004 | Harbour seal | 2018-10-05 | Male | - | Germany | North Sea | Shot | + | + | SP, IL* |
| PV-GE-005 | Harbour seal | 2018-12-03 | Female | Adult | Germany | North Sea | Found dead | + | + | SP, IL* |
| PV-GE-006 | Harbour seal | 2018-12-08 | Female | Adult | Germany | North Sea | Found dead | + | + | SP, IL* |
| PV-GE-007 | Harbour seal | 2018-12-09 | Female | Adult | Germany | North Sea | Found dead | + | + | SP, IL* |
| PV-GE-008 | Harbour seal | 2018-12-09 | Female | Adult | Germany | North Sea | Found dead | + | + | SP, IL* |
| PV-GE-009 | Harbour seal | 2018-12-09 | Female | Adult | Germany | North Sea | Found dead | + | + | SP, IL* |
| PV-GE-010 | Harbour seal | 2018-12-10 | Female | Adult | Germany | North Sea | Found dead | + | + | SP, IL* |
| PV-GE-011 | Harbour seal | 2018-12-11 | Female | Adult | Germany | North Sea | Found dead | + | + | SP, IL* |
| PV-GE-012 | Harbour seal | 2018-12-30 | Female | Adult | Germany | North Sea | Found dead | + | + | SP, IL* |
| PV-SE-018 | Harbour seal | 2018-10-01 | - | - | Sweden | Skagerarak | Shot | | + | IL |
| PV-SE-019 | Harbour seal | 2018-10-12 | Female | Adult | Sweden | Skagerrak | Shot | + | + | SP, IL |
| PV-SE-020 | Harbour seal | 2018-12-25 | Female | Adult | Sweden | Skagerrak | Shot | + | | SP |
| PV-DK-043 | Harbour seal | 2018-10-10 | Male | Juvenile | Denmark | The Sound | Shot | | + | PL |

| Sample number | Species | Collection date | Sex | Age | Country | Area | Origin of animal | Swab | Tissue | Extraction |
|---|---|---|---|---|---|---|---|---|---|---|
| PV-DK-044 | Harbour seal | 2018-10-10 | - | - | Denmark | The Sound | NA | | + | PL |
| PV-DK-045 | Harbour seal | 2019-07-21 | Male | Last year | Denmark | Danish Straits | Bycaught | + | | SP |
| PV-DK-046 | Harbour seal | 2019-08-11 | Female | Current year | Denmark | Danish Straits | Bycaught | + | + | SP, IL |
| PV-DK-047 | Harbour seal | 2019-08-28 | Male | Last year | Denmark | Danish Straits | Bycaught | + | + | SP, IL |
| PV-DK-048 | Harbour seal | 2019-09-18 | Female | Current year | Denmark | Danish Straits | Shot | + | + | SP, IL |
| PV-DK-049 | Harbour seal | 2019-10-09 | Male | Last year | Denmark | Danish Straits | Shot | + | + | SP, IL |
| PV-DK-050 | Harbour seal | 2019-10-22 | Female | Last year | Denmark | Danish Straits | Bycaught | + | + | SP, IL |
| PV-DK-051 | Harbour seal | 2019-04-28 | Male | Last year | Denmark | Kattegat | Bycaught | + | + | SP, IL |
| PV-DK-052 | Harbour seal | 2019-04-28 | Female | Last year | Denmark | Kattegat | Shot | + | + | SP, IL |
| PV-GE-013 | Harbour seal | 2019-01-04 | Male | Last year | Germany | North Sea | Found dead | + | | SP |
| PV-GE-014 | Harbour seal | 2019-01-07 | Female | Adult | Germany | North Sea | Found dead | + | + | SP, IL* |
| PV-GE-015 | Harbour seal | 2019-01-08 | Female | Adult | Germany | North Sea | Shot | + | | SP |
| PV-GE-016 | Harbour seal | 2019-03-10 | Female | Last year | Germany | North Sea | Shot | | + | IL |
| PV-GE-017 | Harbour seal | 2019-07-02 | Male | Last year | Germany | North Sea | Found dead | + | + | SP, IL |
| PV-GE-018 | Harbour seal | 2019-07-23 | Male | Last year | Germany | North Sea | Found dead | + | + | SP, IL |
| PV-GE-019 | Harbour seal | 2019-12-05 | Female | Adult | Germany | North Sea | Found dead | + | + | SP, IL* |
| PV-DK-053 | Harbour seal | 2019-04-07 | Male | Last year | Denmark | The Sound | Shot | + | + | SP, IL |
| PV-DK-054 | Harbour seal | 2019-05-15 | Female | Adult | Denmark | The Sound | Shot | + | + | SP, IL |
| PV-DK-055 | Harbour seal | 2019-05-24 | Male | Current year | Denmark | The Sound | Shot | + | + | SP, IL |
| PV-DK-056 | Harbour seal | 2019-06-03 | Male | Last year | Denmark | The Sound | Shot | + | + | SP, IL |
| PV-DK-057 | Harbour seal | 2019-06-03 | Female | Adult | Denmark | The Sound | Shot | + | + | SP, IL |
| PV-DK-058 | Harbour seal | 2019-08-19 | Female | Current year | Denmark | The Sound | Bycaught | + | + | SP, IL |
| PV-DK-059 | Harbour seal | 2019-08-24 | Male | Adult | Denmark | The Sound | Shot | + | | SP, IL |
| PV-GE-020 | Harbour seal | 2019-03-14 | Male | - | Germany | North Sea | Shot | | + | IL |

**Supplementary Table S2.** Overview of harbour seal samples screened in this study including sample information such as collection date, sex, age, collection area and sample type listed when possible. (-) indicates that there is no available information. The sample type for each extraction is indicated by SP = Pooled extractions of tracheal swabs, IL = Individual extraction from lung tissue, PL = Pooled extractions of lung tissue from 3-5 individuals. * = Samples which were screened separately possibly as pooled samples.

**Supplementary Table S3**

| Sample number | Species | Collection date | Sex | Age | Country | Area | Origin of animal | Swab | Tissue | Extraction |
|---|---|---|---|---|---|---|---|---|---|---|
| PH-SE-001 | Ringed seal | 2002-04-29 | Female | 2 | Sweden | Baltic proper | Bycaught | | + | PL |
| PH-SE-002 | Ringed seal | 2002-09-30 | Female | 0-1 | Sweden | Baltic proper | Bycaught | | + | PL |
| PH-SE-003 | Ringed seal | 2002-09-22 | Male | 0-1 | Sweden | Bothnian Sea | Bycaught | | + | PL |
| PH-SE-004 | Ringed seal | 2002-09-24 | Male | - | Sweden | Bothnian Sea | Bycaught | | + | PL |
| PH-SE-005 | Ringed seal | 2003-06-26 | Female | - | Sweden | Bothnian Bay | Bycaught | | + | PL |
| PH-SE-006 | Ringed seal | 2003-05-05 | Male | 0-1 | Sweden | Bothnian Sea | Bycaught | | + | PL |
| PH-SE-007 | Ringed seal | 2003-08-22 | Female | - | Sweden | Bothnian Sea | Bycaught | | + | PL |
| PH-SE-008 | Ringed seal | 2003-10-13 | Male | - | Sweden | Bothnian Sea | Bycaught | | + | PL |
| PH-SE-009 | Ringed seal | 2006-10-25 | Male | 0-1 | Sweden | Baltic proper | Bycaught | | + | PL |
| PH-SE-010 | Ringed seal | 2006-10-11 | Female | 0-1 | Sweden | Bornholm Basin | Bycaught | | + | PL |
| PH-SE-011 | Ringed seal | 2006-12-13 | Male | - | Sweden | Bothnian Sea | Bycaught | | + | PL |
| PH-SE-012 | Ringed seal | 2007-09-19 | Female | Adult | Sweden | Bothnian Bay | Found dead | | + | PL |
| PH-SE-013 | Ringed seal | 2007-05-11 | Male | 0-1 | Sweden | Bothnian Sea | Bycaught | | + | PL |
| PH-SE-014 | Ringed seal | 2007-06-26 | Male | - | Sweden | Bothnian Sea | Found alive | | + | PL |
| PH-SE-015 | Ringed seal | 2007-10-02 | Male | - | Sweden | Bothnian Sea | Bycaught | | + | PL |
| PH-SE-016 | Ringed seal | 2007-11-05 | Female | 1 | Sweden | Bothnian Sea | Bycaught | | + | PL |
| PH-SE-017 | Ringed seal | 2008-05-23 | Male | 12 | Sweden | Bothnian Bay | Shot | | + | PL |
| PH-SE-018 | Ringed seal | 2008-05-28 | Female | 13 | Sweden | Bothnian Bay | Shot | | + | PL |
| PH-SE-019 | Ringed seal | 2008-05-29 | Female | 7 | Sweden | Bothnian Bay | Shot | | + | PL |
| PH-SE-020 | Ringed seal | 2008-06-16 | Male | 3 | Sweden | Bothnian Bay | Shot | | + | PL |
| PH-SE-021 | Ringed seal | 2008-04-06 | Male | 0-1 | Sweden | Bothnian Sea | Entanglement | | + | PL |
| PH-SE-022 | Ringed seal | 2008-05-20 | Female | 0-1 | Sweden | Bothnian Sea | Entanglement | | + | PL |
| PH-SE-023 | Ringed seal | 2008-08-02 | Female | 0-1 | Sweden | Bothnian Sea | Entanglement | | + | PL |
| PH-SE-024 | Ringed seal | 2013-04-22 | Male | 11 | Sweden | Baltic proper | Shot | | + | PL |
| PH-SE-025 | Ringed Seal | 2013-08-27 | Female | 0-1 | Sweden | Bothnian Bay | Entanglement | | + | PL |

| Sample number | Species | Collection date | Sex | Age | Country | Area | Origin of animal | Swab | Tissue | Extraction |
|---|---|---|---|---|---|---|---|---|---|---|
| PH-SE-026 | Ringed seal | 2013-11-01 | Female | 3 | Sweden | Bothnian Bay | Shot | | + | PL |
| PH-SE-027 | Ringed seal | 2013-11-02 | Male | 12 | Sweden | Bothnian Bay | Shot | | + | PL |
| PH-SE-028 | Ringed seal | 2013-08-02 | Male | 14 | Sweden | Bothnian Sea | Found alive | | + | PL |
| PH-SE-029 | Ringed seal | 2013-11-19 | Male | 3 | Sweden | Bothnian Sea | Entanglement | | + | PL |
| PH-SE-030 | Ringed seal | 2014-09-28 | Male | 0-1 | Sweden | Baltic proper | Entanglement | | + | PL |
| PH-SE-031 | Ringed seal | 2014-06-20 | Male | 0-1 | Sweden | Bothnian Bay | Entanglement | | + | PL |
| PH-SE-032 | Ringed seal | 2014-10-17 | Male | 1 | Sweden | Bothnian Bay | Entanglement | | + | PL |
| PH-SE-033 | Ringed seal | 2014-11-03 | Female | 23 | Sweden | Bothnian Bay | Shot | | + | PL |
| PH-SE-034 | Ringed seal | 2014-04-02 | Male | 0-1 | Sweden | Bothnian Sea | Found dead | | + | PL |
| PH-SE-035 | Ringed seal | 2014-05-26 | Male | 0-1 | Sweden | Bothnian Sea | Entanglement | | + | PL |
| PH-SE-036 | Ringed seal | 2014-06-28 | Female | 0-1 | Sweden | Bothnian Sea | Entanglement | | + | PL |
| PH-SE-037 | Ringed seal | 2015-10-09 | Female | 0-1 | Sweden | Baltic proper | Entanglement | | + | PL |
| PH-SE-038 | Ringed seal | 2015-05-30 | Male | 29 | Sweden | Bothnian Bay | Shot | | + | PL |
| PH-SE-039 | Ringed seal | 2015-06-22 | Female | 1 | Sweden | Bothnian Bay | Shot | | + | PL |
| PH-SE-040 | Ringed seal | 2015-11-19 | Female | 10 | Sweden | Bothnian Bay | Shot | | + | PL |
| PH-SE-041 | Ringed seal | 2015-12-13 | Male | 5 | Sweden | Bothnian Bay | Shot | | + | PL |
| PH-SE-042 | Ringed seal | 2015-04-15 | Female | 3 | Sweden | Bothnian Sea | Shot | | + | PL |
| PH-SE-043 | Ringed seal | 2015-05-27 | Female | 0-1 | Sweden | Bothnian Sea | Entanglement | | + | PL |
| PH-SE-044 | Ringed seal | 2017-05-06 | Male | 18 | Sweden | Bothnian Bay | Shot | | + | PL |
| PH-SE-045 | Ringed seal | 2017-08-01 | Female | 9 | Sweden | Bothnian Bay | Shot | | + | PL |
| PH-SE-046 | Ringed seal | 2017-08-21 | Male | 8 | Sweden | Bothnian Bay | Shot | | + | PL |
| PH-SE-047 | Ringed seal | 2017-09-24 | Male | 5 | Sweden | Bothnian Bay | Shot | | + | PL |
| PH-SE-048 | Ringed seal | 2017-10-01 | Male | 26 | Sweden | Bothnian Bay | Shot | | + | PL |
| PH-SE-049 | Ringed seal | 2017-10-05 | Female | 17 | Sweden | Bothnian Bay | Shot | | + | PL |
| PH-SE-050 | Ringed seal | 2017-10-22 | Female | 11 | Sweden | Bothnian Bay | Shot | | + | PL |

| Sample number | Species | Collection date | Sex | Age | Country | Area | Origin of animal | Swab | Tissue | Extraction |
|---|---|---|---|---|---|---|---|---|---|---|
| PH-SE-051 | Ringed seal | 2017-10-29 | Female | 11 | Sweden | Bothnian Bay | Shot | | + | PL |
| PH-SE-052 | Ringed seal | 2017-11-05 | Female | 0-1 | Sweden | Bothnian Bay | Shot | | + | PL |
| PH-SE-053 | Ringed seal | 2017-06-03 | Male | 0-1 | Sweden | Bothnian Sea | Entanglement | | + | PL |
| PH-SE-054 | Ringed seal | 2017-10-20 | Female | 0-1 | Sweden | Bothnian Sea | Entanglement | | + | IL |
| PH-SE-055 | Ringed seal | 2017-12-18 | Male | 0-1 | Sweden | Bothnian Sea | Shot | | + | PL |
| PH-SE-056 | Ringed seal | 2018-03-15 | Male | 23 | Sweden | Baltic proper | Found dead | | + | PL |
| PH-SE-057 | Ringed seal | 2018-06-12 | Female | - | Sweden | Bothnian Bay | Shot | + | + | SP, PL |
| PH-SE-058 | Ringed seal | 2018-06-13 | Male | - | Sweden | Bothnian Bay | Shot | + | + | SP, PL |
| PH-SE-059 | Ringed seal | 2018-06-14 | Male | - | Sweden | Bothnian Bay | Shot | + | + | SP, PL |
| PH-SE-060 | Ringed seal | 2018-06-14 | Female | - | Sweden | Bothnian Bay | Shot | + | + | SP, PL |
| PH-SE-061 | Ringed seal | 2018-06-14 | Female | - | Sweden | Bothnian Bay | Shot | + | + | SP, PL |
| PH-SE-062 | Ringed seal | 2018-10-18 | Male | - | Sweden | Bothnian Bay | Drowning | + | + | SP, IL |
| PH-SE-063 | Ringed seal | 2018-09-02 | Male | - | Sweden | Danish straits | Found dead | | + | IL |
| PH-SE-064 | Ringed seal | 2019-06-04 | Male | - | Sweden | Bothnian Bay | Shot | + | + | SP, IL* |
| PH-SE-065 | Ringed seal | 2019-06-05 | Female | - | Sweden | Bothnian Bay | Shot | + | + | SP, IL* |
| PH-SE-066 | Ringed seal | 2019-06-05 | Male | - | Sweden | Bothnian Bay | Shot | + | + | SP, IL* |
| PH-SE-067 | Ringed seal | 2019-06-05 | Male | - | Sweden | Bothnian Bay | Shot | + | + | SP, IL* |
| PH-SE-068 | Ringed seal | 2019-06-06 | Female | - | Sweden | Bothnian Bay | Shot | + | + | SP, IL* |
| PH-SE-069 | Ringed seal | 2019-06-07 | Female | - | Sweden | Bothnian Bay | Shot | | + | SP, IL* |
| PH-SE-070 | Ringed seal | 2019-06-07 | Female | - | Sweden | Bothnian Bay | Shot | | + | SP, IL* |
| PH-SE-071 | Ringed seal | 2019-06-07 | Female | - | Sweden | Bothnian Bay | Shot | + | + | SP, IL* |
| PH-SE-072 | Ringed seal | 2019-06-07 | Male | - | Sweden | Bothnian Bay | Shot | + | + | SP, IL* |
| PH-SE-073 | Ringed seal | 2019-06-07 | Female | - | Sweden | Bothnian Bay | Shot | + | + | SP, IL* |

**Supplementary Table S3.** Overview of ringed seal samples screened in this study including sample information such as collection date, sex, age, collection area and sample type listed when possible. (-) indicates that there is no available information. The sample type for each extraction is indicated by SP = Pooled extractions of tracheal swabs, IL = Individual extraction from lung tissue, PL = Pooled extractions of

lung tissue from 3-5 individuals and PMO = Pooled extractions of mixed organs (lung, spleen, mesenteric lymph node, liver and brain) from the same individual. * = Samples which were screened separately possibly as pooled samples.

**Supplementary Table S4.**

| Sample number | Species | Collection date | Sex | Age | Country | Area | Origin of animal | Swab | Tissue | Extraction |
|---|---|---|---|---|---|---|---|---|---|---|
| PP-DK-001 | Harbour porpoise | 2007-05-22 | Male | Adult | Denmark | Kattegat | Found dead | | + | IL |
| PP-DK-002 | Harbour porpoise | 2007-04-10 | Male | Immature | Denmark | Zealand | Bycaught | | + | PL |
| PP-DK-003 | Harbour porpoise | 2010-01-05 | Male | - | Denmark | Danish straits | Found dead | | + | PL |
| PP-DK-004 | Harbour porpoise | 2010-05-13 | Male | - | Denmark | Danish straits | Bycaught | | + | PL |
| PP-DK-005 | Harbour porpoise | 2010-03-09 | Male | Immature | Denmark | Kattegat | Bycaught | | + | IL |
| PP-DK-006 | Harbour porpoise | 2010-04-07 | Male | - | Denmark | Kattegat | Bycaught | | + | PL |
| PP-DK-007 | Harbour porpoise | 2010-05-02 | Male | - | Denmark | Kattegat | Bycaught | | + | PL |
| PP-DK-008 | Harbour porpoise | 2010-05-28 | Male | - | Denmark | Kattegat | Bycaught | | + | PL |
| PP-DK-009 | Harbour porpoise | 2010-06-03 | Female | - | Denmark | Kattegat | Bycaught | | + | IL |
| PP-DK-010 | Harbour porpoise | 2010-06-19 | Female | - | Denmark | Kattegat | Bycaught | | + | IL |
| PP-DK-011 | Harbour porpoise | 2010-12-04 | Male | - | Denmark | Skagerrak | Bycaught | | + | IL |
| PP-DK-012 | Harbour porpoise | 2010-03-15 | Female | - | Denmark | The Sound | Bycaught | | + | PL |
| PP-DK-013 | Harbour porpoise | 2010-03-15 | Female | - | Denmark | The Sound | Bycaught | | + | PL |
| PP-DK-014 | Harbour porpoise | 2010-04-15 | Male | - | Denmark | The Sound | Found dead | | + | PL |
| PP-DK-015 | Harbour porpoise | 2010-04-22 | Male | - | Denmark | The Sound | Bycaught | | + | PL |
| PP-DK-016 | Harbour porpoise | 2010-04-22 | Male | - | Denmark | The Sound | Bycaught | | + | PL |
| PP-DK-017 | Harbour porpoise | 2010-04-29 | Female | - | Denmark | The Sound | Bycaught | | + | PL |
| PP-DK-018 | Harbour porpoise | 2010-06-04 | Male | Immature | Denmark | The Sound | Bycaught | | + | IL |
| PP-DK-019 | Harbour porpoise | 2010-06-04 | Male | Immature | Denmark | The Sound | Bycaught | | + | IL |
| PP-DK-020 | Harbour porpoise | 2010-06-08 | Male | Immature | Denmark | The Sound | Bycaught | | + | IL |
| PP-DK-021 | Harbour porpoise | 2010-07-14 | Male | - | Denmark | The Sound | Bycaught | | + | PL |
| PP-DK-022 | Harbour porpoise | 2010-07-22 | Female | - | Denmark | The Sound | Bycaught | | + | IL |
| PP-DK-023 | Harbour porpoise | 2010-08-05 | Female | - | Denmark | The Sound | Drowned | | + | IL |
| PP-DK-024 | Harbour porpoise | 2011-08-05 | Female | - | Denmark | Danish Straits | Bycaught | | + | IL |

| Sample number | Species | Collection date | Sex | Age | Country | Area | Origin of animal | Swab | Tissue | Extraction |
|---|---|---|---|---|---|---|---|---|---|---|
| PP-DK-025 | Harbour porpoise | 2011-07-02 | Female | Adult | Denmark | Kattegat | Bycaught? | | + | IL |
| PP-DK-026 | Harbour porpoise | 2011-02-04 | Female | Juvenile | Denmark | Zealand | Bycaught | | + | IL |
| PP-DK-027 | Harbour porpoise | 2012-07-02 | Female | - | Denmark | North Sea | Found dead | | + | IL |
| PP-DK-028 | Harbour porpoise | 2015-09-10 | Male | 0 years | Denmark | The Sound | Found dead | | + | IL |
| PP-DK-029 | Harbour porpoise | 2017-09-28 | Female | 0 years | Denmark | Danish straits | Found dead | | + | IL |
| PP-SE-001 | Harbour porpoise | 2017-11-20 | Male | Adult | Sweden | The Sound | Found dead, pneumea | | + | IL |
| PP-DK-030 | Harbour porpoise | 2018-04-05 | Male | - | Denmark | Danish straits | Found dead in pound net | | + | IL |
| PP-DK-031 | Harbour porpoise | 2018-04-19 | Male | - | Denmark | Danish straits | Bycaught | | + | IL |
| PP-GE-001 | Harbour porpoise | 2018-08-01 | Male | - | Germany | Danish Straits | Bycaught? | + | + | SP, IL |
| PP-DK-032 | Harbour porpoise | 2018-09-01 | Female | Neonate | Denmark | Danish Straits | Found dead | + | + | SP, IL |
| PP-GE-002 | Harbour porpoise | 2018-12-01 | Male | - | Germany | Danish Straits | Bycaught | + | + | SP*, IL |
| PP-SE-002 | Harbour porpoise | 2018-12-18 | Male | Adult | Sweden | Kattegat (Skåne) | Found dead | | + | IL |
| PP-SE-003 | Harbour porpoise | 2018-03-14 | Female | Subadult | Sweden | The Sound | Entanglement | | + | IL |
| PP-SE-004 | Harbour porpoise | 2018-04-08 | Male | Subadult | Sweden | The Sound | Found dead | | + | IL |
| PP-DK-033 | Harbour porpoise | 2019-09-05 | Male | Adult | Denmark | Bornholm Basin | Found dead | + | + | SP, IL |
| PP-GE-003 | Harbour porpoise | 2019-02-25 | Female | - | Germany | Danish Straits | Found dead | + | + | SP, IL |
| PP-DK-034 | Harbour porpoise | 2019-07-27 | Female | Neonate | Denmark | Danish Straits | Found dead | | + | IL |
| PP-GE-004 | Harbour porpoise | 2019-08-08 | Male | - | Germany | Danish Straits | Bycaught | + | + | SP, IL |
| PP-GE-005 | Harbour porpoise | 2019-08-10 | Female | Adult | Germany | Danish Straits | Found dead | | + | IL |
| PP-GE-006 | Harbour porpoise | 2019-08-11 | Female | Neonat | Germany | Danish Straits | Found dead | | + | PMO |
| PP-GE-007 | Harbour porpoise | 2019-08-14 | Female | Juvenile | Germany | Danish Straits | Bycaught? | | + | PMO |
| PP-DK-035 | Harbour porpoise | 2019-08-22 | Male | Neonate | Denmark | Danish Straits | Found dead | + | + | SP, IL |
| PP-GE-008 | Harbour porpoise | 2019-08-24 | Male | - | Germany | Danish Straits | Bycaught | + | + | SP, IL |
| PP-GE-009 | Harbour porpoise | 2019-09-07 | Female | Juvenile | Germany | Danish Straits | Found dead | | + | PMO |

| Sample number | Species | Collection date | Sex | Age | Country | Area | Origin of animal | Swab | Tissue | Extraction |
|---|---|---|---|---|---|---|---|---|---|---|
| PP-GE-010 | Harbour porpoise | 2019-09-10 | Female | Adult | Germany | Danish Straits | Bycaught? | + | + | SP, IL |
| PP-GE-011 | Harbour porpoise | 2019-09-17 | Male | Neonat | Germany | Danish Straits | Bycaught | + | + | SP, IL |
| PP-GE-012 | Harbour porpoise | 2019-09-19 | Female | Juvenile | Germany | Danish Straits | Found dead | | + | PMO |
| PP-GE-013 | Harbour porpoise | 2019-09-19 | Male | Neonat | Germany | Danish Straits | Found dead | | + | PMO |
| PP-GE-014 | Harbour porpoise | 2019-09-21 | Male | Juvenile | Germany | Danish Straits | Found dead | | + | PMO |
| PP-GE-015 | Harbour porpoise | 2019-09-23 | Male | Juvenile | Germany | Danish Straits | Bycaught? | | + | PMO |
| PP-DK-036 | Harbour porpoise | 2019-09-27 | Male | Juvenile | Denmark | Danish Straits | Found dead | + | + | SP, IL |
| PP-GE-016 | Harbour porpoise | 2019-09-27 | Male | Juvenile | Germany | Danish Straits | Found dead | | + | PMO |
| PP-GE-017 | Harbour porpoise | 2019-09-29 | Female | Juvenile | Germany | Danish Straits | Found dead | | + | PMO |
| PP-GE-018 | Harbour porpoise | 2019-10-02 | Male | Juvenile | Germany | Danish Straits | Found dead | + | + | SP, IL |
| PP-GE-019 | Harbour porpoise | 2019-10-02 | Male | Neonat | Germany | Danish Straits | Bycaught? | + | + | SP, IL |
| PP-GE-020 | Harbour porpoise | 2019-10-02 | Male | Juvenile | Germany | Danish Straits | Found dead | | + | PMO |
| PP-GE-021 | Harbour porpoise | 2019-10-04 | Female | Juvenile | Germany | Danish Straits | Found dead | | + | PMO |
| PP-GE-022 | Harbour porpoise | 2019-10-13 | Female | Juvenile | Germany | Danish Straits | Bycaught? | | + | PMO |
| PP-GE-023 | Harbour porpoise | 2019-10-14 | Female | Adult | Germany | Danish Straits | Found dead | | + | PMO |
| PP-GE-024 | Harbour porpoise | 2019-10-21 | Male | Juvenile | Germany | Danish Straits | Bycaught? | | + | PMO |
| PP-GE-025 | Harbour porpoise | 2019-10-30 | Female | Juvenile | Germany | Danish Straits | Found dead | | + | PMO |
| PP-GE-026 | Harbour porpoise | 2019-11-05 | Female | Juvenile | Germany | Danish Straits | Found dead | | + | PMO |
| PP-GE-027 | Harbour porpoise | 2019-11-22 | Female | Juvenile | Germany | Danish Straits | Bycaught? | | + | PMO |
| PP-GE-028 | Harbour porpoise | 2019-11-24 | Female | Adult | Germany | Danish Straits | Found dead | | + | PMO |
| PP-GE-029 | Harbour porpoise | 2019-12-17 | Female | Juvenile | Germany | Danish Straits | Bycaught | | + | PMO |
| PP-GE-030 | Harbour porpoise | 2019-10-12 | Female | Adult | Germany | German Baltic | Found dead | | + | PMO |
| PP-DK-037 | Harbour porpoise | 2019-03-06 | Female | Juvenile | Denmark | Kattegat | Bycaught | + | + | SP, IL |
| PP-DK-038 | Harbour porpoise | 2019-05-23 | Male | Juvenile | Denmark | Kattegat | Found dead | + | + | SP, IL |

| Sample number | Species | Collection date | Sex | Age | Country | Area | Origin of animal | Swab | Tissue | Extraction |
|---|---|---|---|---|---|---|---|---|---|---|
| PP-DK-039 | Harbour porpoise | 2019-07-20 | Female | Juvenile | Denmark | Kattegat | Found dead | + | + | SP, IL |
| PP-DK-040 | Harbour porpoise | 2019-10-02 | Male | Juvenile | Denmark | Kattegat | Bycaught? | + | + | SP, IL |
| PP-GE-031 | Harbour porpoise | 2019-04-11 | Male | Juvenile | Germany | North Sea | Found dead | + | | SP |
| PP-GE-032 | Harbour porpoise | 2019-04-16 | Male | Juvenile | Germany | North Sea | Found dead | + | + | SP, IL |
| PP-GE-033 | Harbour porpoise | 2019-09-22 | Male | Neonat | Germany | North Sea | Found dead | + | + | SP, IL |
| PP-DK-041 | Harbour porpoise | 2019-03-06 | Male | Juvenile | Denmark | The Sound | Bycaught | | + | IL |

**Supplementary Table S4.** Overview of harbour porpoise samples screened in this study including sample information such as collection date, sex, age, collection area and sample type listed when possible. (-) indicates that there is no available information. The sample type for each extraction is indicated by SP = Pooled extractions of tracheal swabs, IL = Individual extraction from lung tissue, PL = Pooled extractions of lung tissue from 3-5 individuals and PMO = Pooled extractions of mixed organs (lung, spleen, mesenteric lymph node, liver and brain) from the same individual. * Indicates one swap (PP-GE-002) taken from the blowhole of a harbour porpoise
